# B cell-intrinsic interleukin 17 receptor A signaling supports the establishment of chronic murine gammaherpesvirus 68 infection

**DOI:** 10.1128/jvi.01842-25

**Published:** 2025-12-03

**Authors:** Sheikh Tahir Majeed, Nicholas P. Huss, Samantha M. Bradford, Christopher N. Jondle

**Affiliations:** 1 Department of Investigative Medicine and Center for Immunobiology, Western Michigan University Homer Stryker M.D. School of Medicine, Kalamazoo, Michigan, USA; University of Toronto, Toronto, Ontario, Canada

**Keywords:** germinal center B cells, B cells, IL-17RA, murine gammaherpesvirus 68, MHV68, gammaherpesvirus

## Abstract

The human-specific gammaherpesviruses Epstein-Barr virus and Kaposi’s sarcoma-associated herpesvirus infect >95% of all adults, establish lifelong infections, and are associated with multiple different cancers, including B-cell lymphomas. These viruses naturally infect B cells and drive a unique and robust germinal center response to establish their latent viral reservoir in memory B cells. The unique and robust nature of the virus-driven germinal center response increases the risk for B-cell transformation, which is why many gammaherpesvirus-associated cancers are derived from germinal center or post-germinal center B cells. We have previously reported that both global and T cell-specific host IL-17RA signaling is proviral in the context of gammaherpesvirus infection by promoting the virus-driven germinal center response and establishment of latent viral infection. In this study, we examine the role of B cell-specific IL-17RA signaling in the context of gammaherpesvirus infection, given these viruses’ reliance on B cells to establish and maintain lifelong infection. Similarly, to what we observed in the context of global and T cell-specific IL-17RA signaling, we found that B cell-intrinsic IL-17RA signaling supports the establishment of viral latency and reactivation in the spleen and peritoneal cavity, as well as promotes the virus-driven germinal center response. Our study reveals the proviral role of B cell-intrinsic IL-17RA signaling in supporting the establishment of chronic gammaherpesvirus infection.

**IMPORTANCE:**

Gammaherpesviruses are lifelong pathogens that are prevalent in over 95% of all adults. These viruses are tumorigenic and associated with multiple cancers, including B-cell lymphomas. They are B-cell tropic viruses that manipulate the germinal center response to establish their latent viral reservoir in memory B cells. This manipulation of the germinal center response is thought to be the target of viral transformation, leading to lymphomagenesis as most gammaherpesvirus-associated cancers are germinal center or post-germinal center derived. In this study, we developed a new mouse model to understand the B cell-intrinsic role of IL-17RA signaling in gammaherpesvirus infection. Previous studies have shown that IL-17RA signaling is proviral in the context of gammaherpesvirus infection, and this study found that while B cell-intrinsic IL-17RA signaling is not the sole factor behind the systemic proviral role of IL-17RA signaling, it plays an important role in the virus-driven germinal center response, the expansion of B-1 B cells, and the establishment of chronic gammaherpesvirus infection.

## INTRODUCTION

Gammaherpesviruses are ubiquitous, DNA viruses that establish lifelong infections in their host. Epstein-Barr virus (EBV) and Kaposi’s Sarcoma-associated herpesvirus (KSHV) are the two human-specific gammaherpesviruses that infect >95% of all adults and are associated with multiple cancers, including B-cell lymphomas (1, 2). These viruses have a biphasic life cycle consisting of lytic replication during the acute phase of infection, followed by lifelong latent infection often characterized as chronic infection (3). The study of chronic EBV and KSHV infection *in vivo* is challenging due to the strict species specificity of gammaherpesviruses. This study utilizes murine gammaherpesvirus 68 (MHV68) as a tractable animal model of gammaherpesvirus infection. MHV68 is a natural rodent pathogen that is genetically, biologically, and pathologically related to EBV and KSHV (3–6). It provides a model system to study chronic gammaherpesvirus infection and pathogenesis in an intact host, while also allowing for the manipulation of host genetics to understand potential host mechanisms that impact gammaherpesvirus infection.

EBV, KSHV, and MHV68 are B-cell-tropic viruses, with EBV and MHV68 establishing a latent viral reservoir in memory B cells by usurping the germinal center response (7–11). These gammaherpesviruses, unlike most other viral infections, usurp B-cell differentiation to establish a latent viral reservoir in memory B cells (7–9). They achieve this by infecting naïve B cells and inducing a germinal center response, which includes both virus-infected and uninfected B cells (7, 12, 13) that subsequently differentiate into class-switched plasma cells, where viral reactivation occurs, or memory B cells that host life-long latent infection (14). The germinal center stage of B-cell differentiation is susceptible to cellular transformation, as germinal center B cells rapidly divide while downregulating tumor suppressors (15) and increasing expression of mutagenic enzymes (16, 17). Consequently, many gammaherpesvirus-driven B-cell lymphomas are of germinal center or post-germinal center origin (18). Furthermore, increased viral reactivation often precedes tumorigenesis (19–22). Importantly, the mechanisms by which gammaherpesviruses induce the germinal center response and factors that promote viral reactivation are poorly understood.

IL-17A is a member of the IL-17 family of cytokines, consisting of IL-17A through IL-17F (23). It signals through the heterodimeric receptor complex of IL-17 receptor A (IL-17RA) and IL-17RC (24–26). The signaling events downstream of the IL-17RA–IL-17RC receptor utilize the adapter Act1 to recruit and ubiquitinate TRAF6, leading to the activation of the NF-kB and MAPK pathways (27–29). Many IL-17RA target genes have NF-kB promoters, and activation of the canonical NF-kB pathway is the primary mediator of inflammatory gene activation downstream of IL-17RA (23, 30). IL-17A plays a diverse role across a number of different conditions. It is critical for the clearance of multiple bacterial and fungal pathogens (31, 32), while also being associated with various autoimmune diseases, such as psoriasis, rheumatoid arthritis, and Crohn’s disease (33). There have been four FDA-approved IL-17 targeting treatments against various inflammatory diseases (34, 35). The role of IL-17A signaling in viral infections is less understood. It inhibits rhinovirus replication, promotes an IFN-γ response against herpes simplex virus 2, and is an important mediator of B-1a B-cell natural antibody response against pulmonary influenza infection (36–38), highlighting an antiviral function of IL-17A signaling. Intriguingly, SARS-CoV-2’s viral protein Orf8 acts as a viral mimic of host IL-17 to activate IL-17RA and IL-17RC (39). Likewise, herpesvirus saimiri, a simian gammaherpesvirus, encodes a viral IL-17, which functions similarly to host IL-17A and IL-17F in cultured cells (40–42). Other gammaherpesviruses, including EBV, KSHV, and MHV68, do not encode a viral IL-17A homolog, yet in the context of infectious mononucleosis, a clinical disease associated with recent EBV infection, there is a significant increase in IL-17A-producing CD4+ T cells that persists at least 1 month following the resolution of clinical symptoms (43). Furthermore, MHV68 infection leads to increased IL-17A production in the lungs during the acute stage of infection and the spleen and peritoneal cavity during the chronic stage of infection by multiple cell types (44, 45). Finally, increases in IL-17A in the lungs following nontypeable *Haemophilus influenzae* secondary infection promote the establishment of MHV68 latency (46). These data suggest that IL-17A signaling may be proviral in the context of some viral infections and that gammaherpesvirus infection induces an IL-17 response.

Global loss of IL-17A signaling during MHV68 infection led to a significant attenuation of viral latency and reactivation in the spleen and peritoneal cavity, as well as a reduction in the viral-driven germinal center response and decreased latent infection in activated/germinal center B cells in the spleen (45). Furthermore, loss of IL-17A signaling during MHV68 infection also impaired the production of irrelevant and self-directed antibody responses, which is a hallmark of EBV and MHV68 infection (47, 48), with the detection of antibodies against horse red blood cells being diagnostic for recent EBV infection (49). Finally, loss of IL-17A signaling specifically in T cells during MHV68 infection resulted in a similar attenuation of viral latency and reactivation as well as the germinal center response in the spleen (50). Together, these data highlight a proviral role for global and more specifically T cell-intrinsic IL-17A signaling during MHV68 infection (51).

IL-17A signaling in B cells in the context of autoimmunity and bacterial infection is known to promote B-cell activation, proliferation, as well as germinal center formation and migration in addition to antibody class switching (52–58). In regard to viral infection, IL-17A signaling in B-1a cells promotes natural antibody production during pulmonary influenza infection (38). Given the significance of B cells to gammaherpesvirus infection (7–13), the role of IL-17A signaling in B cells (38, 52–58), and the noted proviral role for global IL-17RA signaling during gammaherpesvirus infection (45), in this study, we examine the B cell-intrinsic role of IL-17RA signaling during MHV68 infection. B cell-specific IL-17RA deficiency had no impact on B220+ B-cell numbers in naïve animals. Upon infection with MHV68, B cell-specific IL-17RA-deficient mice had significantly attenuated viral latency and reactivation in both the spleen and peritoneal cavity compared to IL-17RA B cell-sufficient control mice. Furthermore, the germinal center response in the infected B cell-specific IL-17RA-deficient mice was significantly decreased compared to the control. Interestingly, the decreased germinal center response in the B cell-specific IL-17RA-deficient mice did not correlate with a reduction in the titers of irrelevant and self-directed antibodies, as was previously observed in the global and T cell-specific IL-17RA-deficient mice (45, 50). This is potentially accounted for by the increase in extrafollicular antibody-secreting B cells observed in the infected B cell-specific IL-17RA-deficient mice, which could compensate for the decrease in other antibody-secreting B cells. Furthermore, IL-17RA signaling in B cells during MHV68 infection supported the expansion of both B-1a and B-1b B cells in the spleen and peritoneal cavity. B-1b B cells are particularly important in the peritoneal cavity for supporting latent infection (59, 60). In summary, our findings show that B cell-intrinsic IL-17RA signaling is proviral in the context of MHV68 infection. It helps promote the virus-driven germinal center response and the infection of B cells, both of which are necessary to establish viral latency. Furthermore, B cell-intrinsic IL-17RA signaling supports viral reactivation. This study, along with our previous ones, indicates that IL-17RA signaling in multiple cell types is critical to fully support the establishment of chronic gammaherpesvirus infection.

## RESULTS

### Mouse model of B cell-specific IL-17RA deficiency

**Fig 1:**
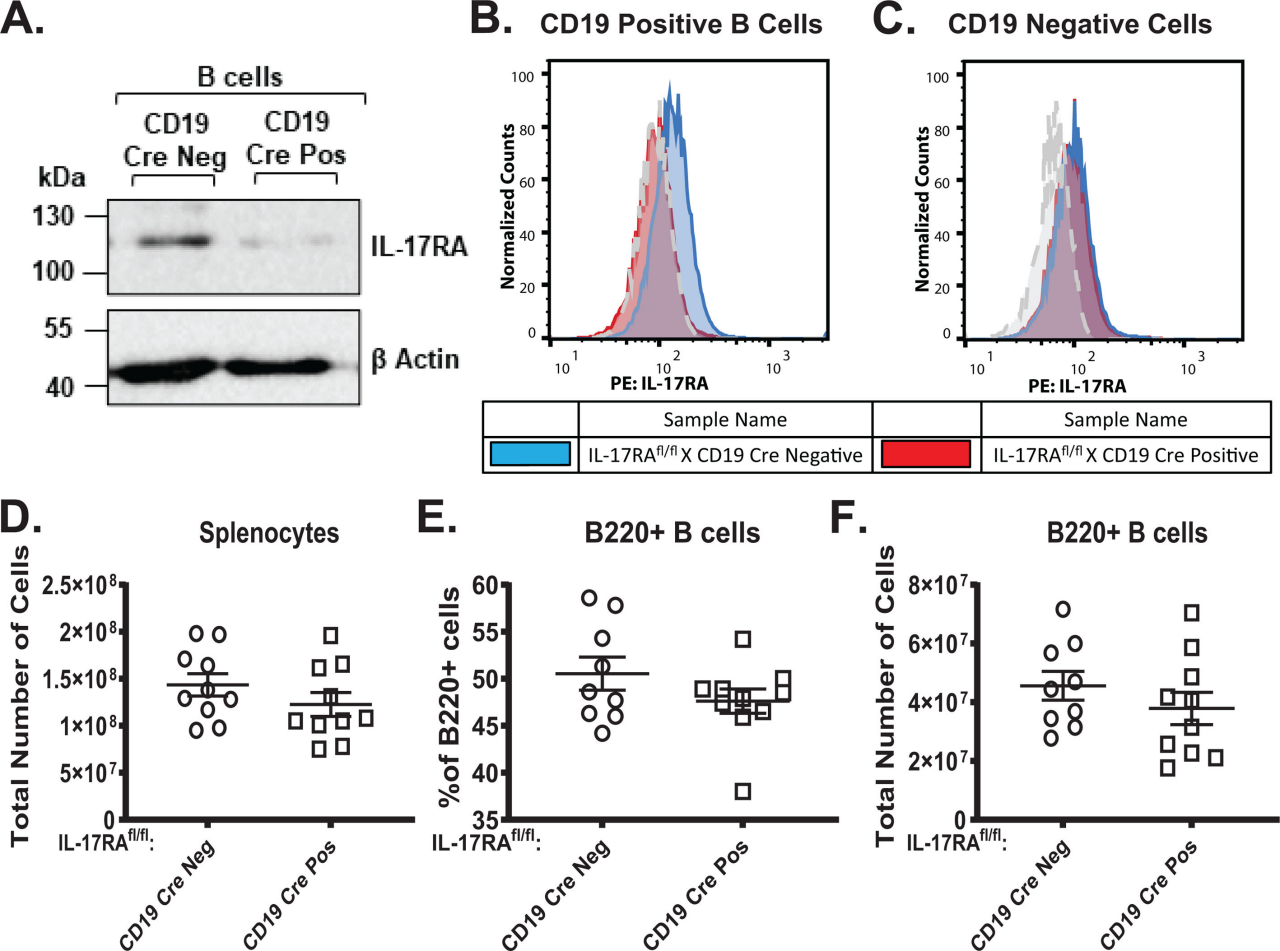
Mouse model of B cell-specific IL-17RA deficiency. Western blot analysis of IL-17RA protein expression in magnetically sorted CD19+ B cells from the spleen of naïve CD19 Cre-negative and CD19 Cre-positive mice (**A**). Cellular expression of IL-17RA in CD19^+^ B cells (**B**) and CD19- non-B cells (**C**) from the spleen of naive CD19 Cre-negative and CD19 Cre-positive littermates was determined by flow cytometry. Gray dashed lines represent FMO controls. Absolute number of splenocytes between CD19 Cre-negative and CD19 Cre-positive naïve mice (**D**). Frequency (**E**) and absolute number (**F**) of B220^+^ B cells in the spleen of CD19 Cre-negative and CD19 Cre-positive naïve littermates. Data are pooled from four to six independent experiments, with each symbol representing an individual mouse.

Having discovered a proviral role of global IL-17RA signaling during gammaherpesvirus infection (45), we next investigated the effect of B cell-intrinsic IL-17RA signaling. B cell-specific IL-17RA deficiency was generated by crossing IL-17RA^fl/fl^ mice (61) to a mouse strain where expression of the Cre recombinase is driven by CD19 (B6.129P2-*Cd19^tm1(cre)Cgn^*/J) (62). Use of the CD19 Cre allows for the recombination of the conditional IL-17RA allele at the earliest stages of B-cell development (63). Successful generation of the B cell-specific IL-17RA-deficient mice was confirmed by examining protein expression of IL-17RA on magnetically sorted CD19-positive B cells from naïve mice (Fig. 1A). Furthermore, expression of IL-17RA in CD19-positive B cells (Fig. 1B) and CD19-negative cells (Fig. 1C) from the spleen of naïve CD19 Cre-negative and CD19 Cre-positive mice was examined via flow cytometry (61). Loss of IL-17RA in B cells had no impact on splenic size (Fig. 1D) nor the frequency (Fig. 1E) or absolute number (Fig. 1F) of B220+ B cells in the spleen of naïve CD19 Cre-positive as compared to CD19 Cre-negative littermates. Similarly, there was no difference in the frequency or absolute number of CD3+ T cells (Fig. S1A and B) and CD11b+ monocytes (Fig. S1C and D) in the spleen of naïve CD19 Cre-positive as compared to CD19 Cre-negative littermates.

### B cell-intrinsic IL-17RA deficiency leads to attenuated establishment of chronic MHV68 infection

**Fig 2:**
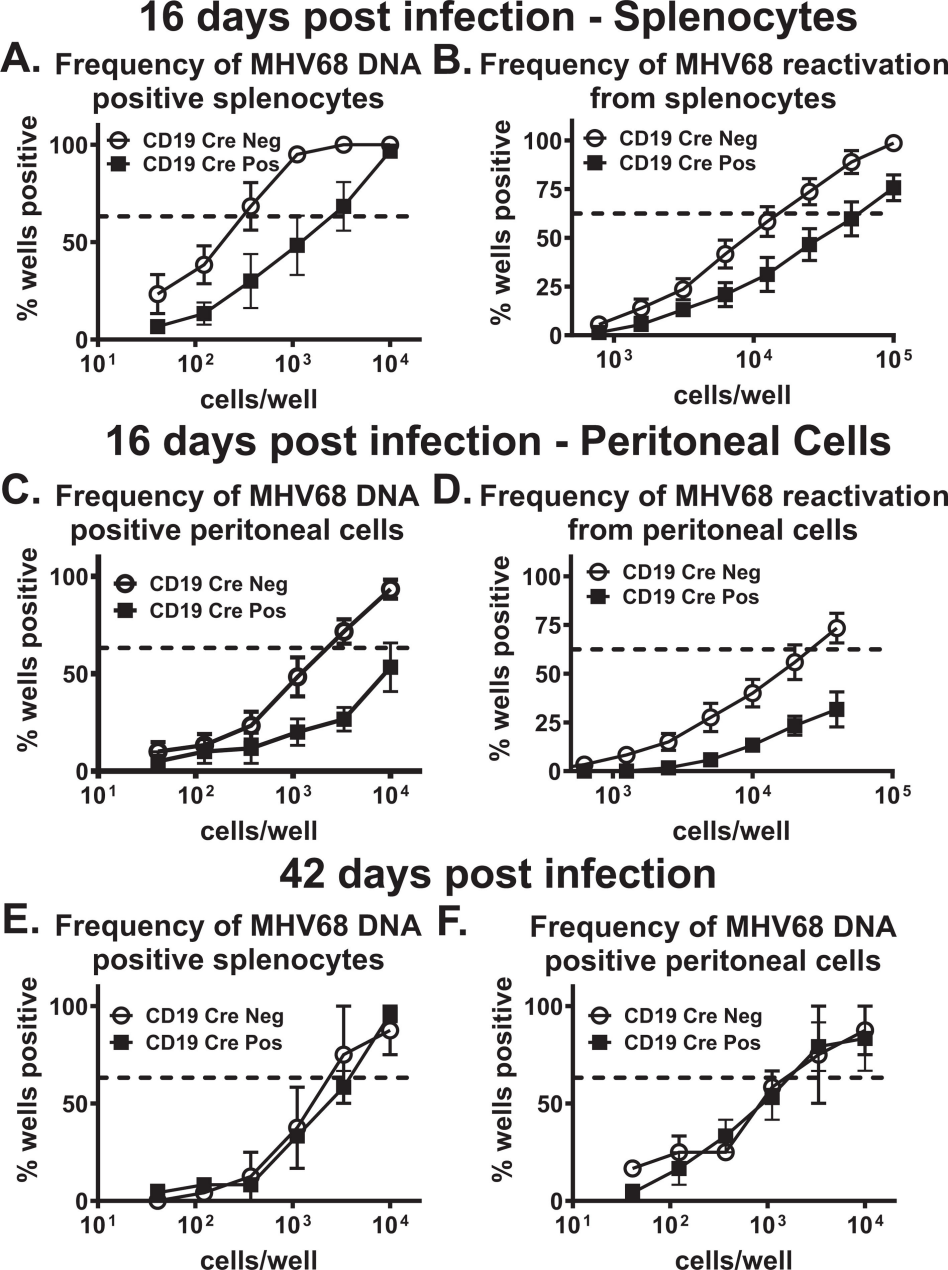
B cell-intrinsic IL-17RA deficiency leads to attenuated establishment of chronic MHV68 infection. CD19 Cre-negative and CD19 Cre-positive mice were intranasally infected with 1,000 PFU of MHV68. Splenocytes and peritoneal cells were collected at 16 and 42 days post-infection and subjected to limiting dilution assays to determine the frequency of MHV68 DNA-positive cells (**A, C, E**) or the frequency of *ex vivo* viral reactivation (**B, D, F**). Each experimental group consists of three to five animals, data were pooled from two to five independent experiments, and SEM is displayed. In the limiting dilution assays, the dotted line is drawn at 63.2%, and the x-coordinate of the intersection of this line with the sigmoid graph represents the inverse of the frequency of positive events.

We previously found that both global and T cell-intrinsic IL-17RA signaling support the establishment of chronic MHV68 infection (45, 50). To determine whether B cell-intrinsic IL-17RA signaling was required for optimal establishment of chronic MHV68 infection, viral latency and reactivation were measured at 16 days post-infection, the peak of viral latency (3), in the spleen and peritoneal cavity of CD19 Cre-positive and CD19 Cre-negative littermates. Consistent with what was observed in the global and T cell-intrinsic IL-17RA-deficient models (45, 50), the frequency of MHV68 DNA-positive splenocytes (Fig. 2A) and the frequency of MHV68 reactivation from splenocytes (Fig. 2B) were significantly attenuated in CD19 Cre-positive mice compared to CD19 Cre-negative littermates. In the peritoneal cavity, the frequency of MHV68 DNA-positive cells (Fig. 2C) and MHV68 reactivation (Fig. 2D) was also attenuated in CD19 Cre-positive mice compared to CD19 Cre-negative littermates. Thus, B cell-intrinsic IL-17RA signaling supports the establishment of chronic MHV68 infection.

Following peak viral latency at 16 days post-infection, the MHV68 latent viral reservoir constricts and stabilizes. This reduction in the latent viral reservoir also contracts the MHV68-driven germinal center response and reduces viral reactivation down to undetectable levels by 42 days post-infection. Similar to what was observed in the global and T cell-intrinsic IL-17RA-deficient models (45, 50), viral reactivation was undetectable in the spleen or peritoneal cavity of CD19 Cre-positive mice and CD19 Cre-negative littermates at 42 days post-infection (data not shown). Furthermore, there was no longer a difference in the frequency of MHV68-infected splenocytes (Fig. 2E) and peritoneal cells (Fig. 2F) between CD19 Cre-positive mice and CD19 Cre-negative littermates at the long-term infection time point of 42 days post-infection. Thus, B cell-intrinsic IL-17RA signaling helps promote peak MHV68 latency at 16 days post-infection but does not impact the long-term maintenance of the latent viral reservoir.

### B cell-intrinsic IL-17RA signaling promotes the MHV68-driven germinal center response during the establishment of latency

**Fig 3:**
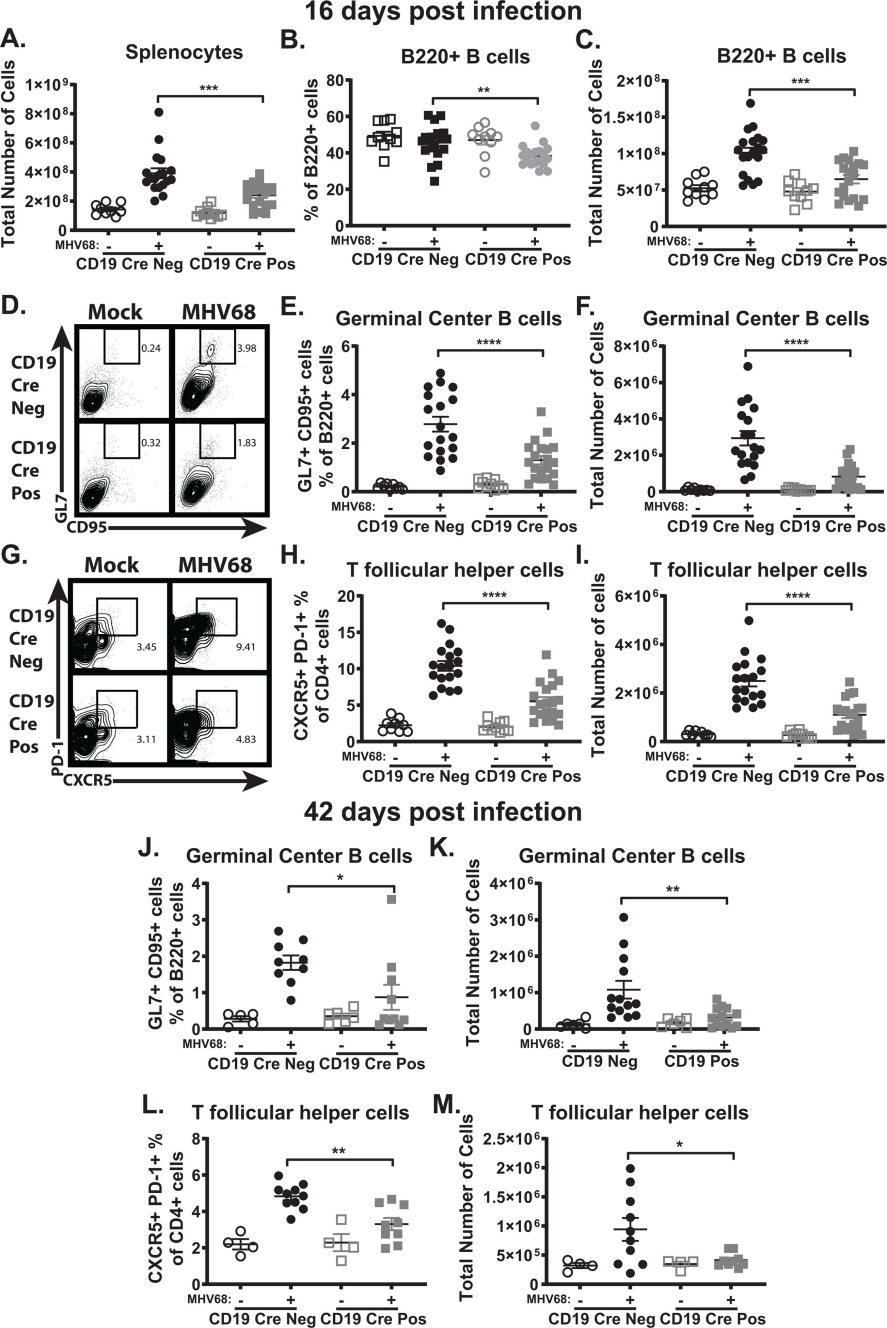
B cell-intrinsic IL-17RA signaling promotes the MHV68-driven germinal center response during the establishment of latency. CD19 Cre-negative and CD19 Cre-positive mice were infected as in Fig. 2. Total splenocytes from naïve and infected CD19 Cre-negative and CD19 Cre-positive mice at 16 days post-infection (**A**). The frequency (**B**) and total number (**C**) of B220+ B cells in the spleen were determined at 16 days post-infection. The germinal center response was measured at 16 and 42 days post-infection in the spleen, with germinal center B cells (**A, B, C, G, H**) defined as B220+GL7+CD95+ cells and T follicular helper cells (**D, E, F, I, J**) defined as CD3+CD4+CXCR5+PD-1+cells. Each experimental group consists of 3–4 animals, and data are pooled from three to five independent experiments. Mean and standard error of the mean are shown. *P < 0.05, **P < 0.01, ****P < 0.0001.

The germinal center response is critical for the establishment of chronic MHV68 infection (7–9). With the attenuation of viral latency observed at 16 days post-infection in CD19 Cre-positive mice compared to CD19 Cre-negative mice (Fig. 2), the germinal center response was investigated next in the spleen. Loss of B cell-intrinsic IL-17RA signaling during MHV68 infection resulted in a decrease in splenomegaly (Fig. 3A), with a significant reduction in the frequency (Fig. 3B) and total number (Fig. 3C) of overall B cells in the spleen of infected CD19 Cre-positive mice compared to CD19 Cre-negative mice. Looking at the germinal center response, there was a reduced frequency and number of germinal center B cells (Fig. 3D through F) and T follicular helper cells (Fig. 3G through I) in CD19 Cre-positive mice compared to CD19 Cre-negative mice at 16 days post-infection. The germinal center B cells can be organized into the dark and light zones of the germinal center, with rapidly proliferating centroblasts making up the dark zone and non-proliferating centrocytes in the light zone (64). Loss of B cell-intrinsic IL-17RA signaling in naïve CD19 Cre-positive mice caused an increase in the frequency of proliferating centroblasts and a subsequent decrease in centrocytes compared to naïve CD19 Cre-positive mice (Fig. S2A and C). That difference in the ratio of centroblasts to centrocytes observed in naïve mice disappeared upon MHV68 infection, with no difference in the frequency of centroblasts (Fig. S2A) and centrocytes (Fig. S2C) between CD19 Cre-positive mice and Cre-negative mice at 16 days post-infection. Figure S3 shows the gating schemes for the determination of all reported cellular frequencies in the spleen.

At the long-term infection time point of 42 days post-infection, the frequency and number of germinal center B cells (Fig. 3J and K) and T follicular helper cells (Fig. 3L and M) were still reduced in CD19 Cre-positive mice compared to CD19 Cre-negative mice. Taken together, these data indicate that B cell-intrinsic IL-17RA signaling during MHV68 infection supports the establishment and maintenance of the virus-driven germinal center response.

### B cell-intrinsic IL-17RA signaling has no effect on the antibody response, including the irrelevant and self-directed class-switched antibodies stimulated by MHV68 infection

**Fig 4:**
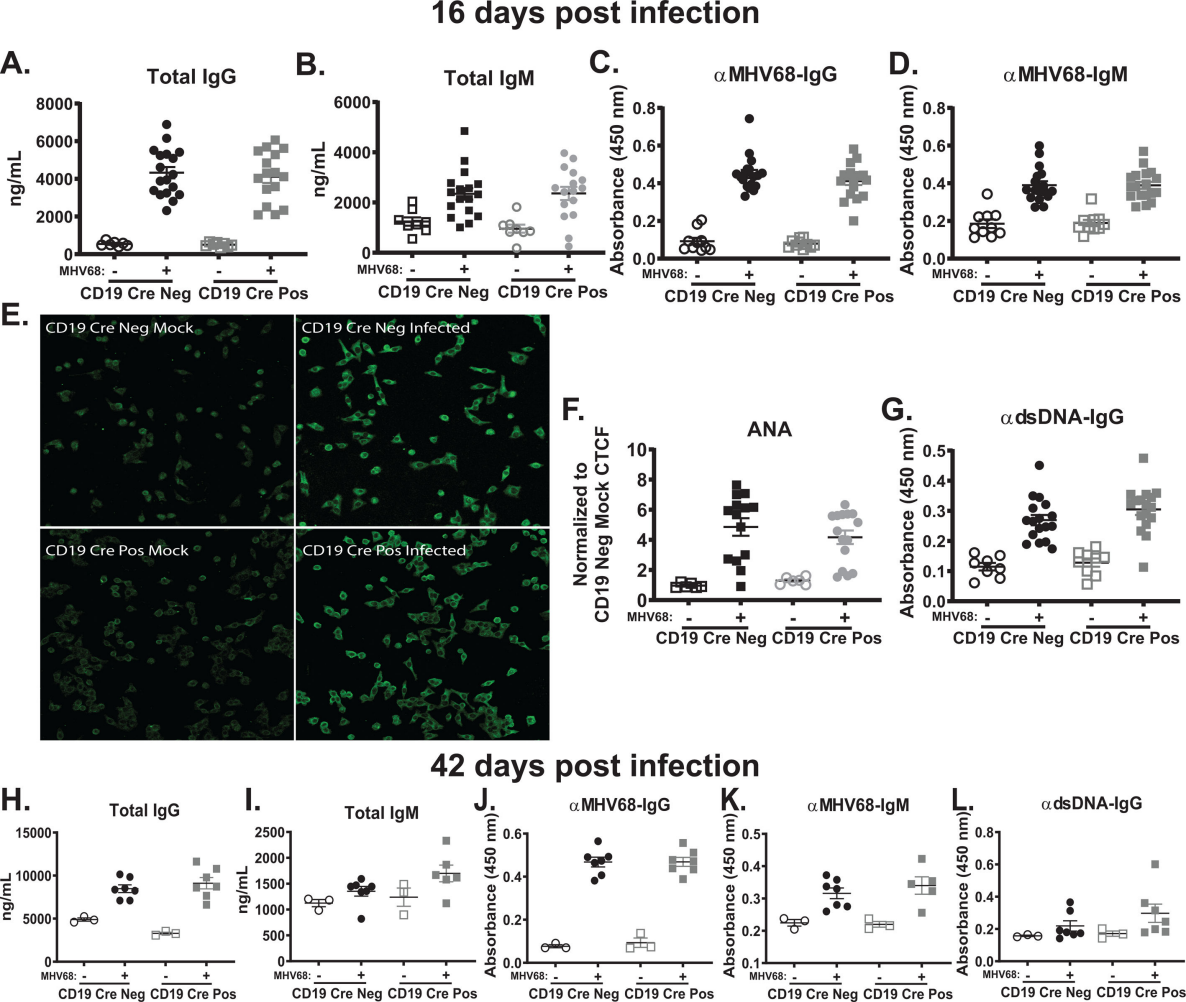
B cell-intrinsic IL-17RA signaling has no effect on the antibody response, including the irrelevant and self-directed class-switched antibodies stimulated by MHV68 infection. CD19 Cre-negative and CD19 Cre-positive mice were infected as in Fig. 2, and serum was collected at 16 and 42 days post-infection. (**A–G**) Sera were used to determine total IgG (**A**) and IgM (**B**), MHV68-specific IgG (**C**) and IgM (**D**), and dsDNA IgG (**G**) antibody titers. (**E**) Reactivity of mouse sera with Hep-2 monolayers (ANA) using anti-mouse IgG FITC-conjugated antibody for detection (Representative images), corrected total cell fluorescence (CTCF) quantified in (**F**). (**H–L**) 42 day post-infection sera were used to determine the total IgG (**H**) and IgM (**I**), MHV68-specific IgG (**J**) and IgM (**K**), and dsDNA IgG (**L**) antibody titers. Data are pooled from two to five independent experiments, with each symbol representing an individual mouse. Mean and standard error of the mean are shown.

Given the decrease in the germinal center response in MHV68-infected CD19 Cre-positive mice (Fig. 3), we next examined the antibody response as an outcome of B-cell differentiation. Unlike what was previously observed by several groups with the global IL-17RA-deficient mice (45, 65) and our group in T cell-specific IL-17RA-deficient mice (50), CD19 Cre-positive mice did not have a baseline increase in IgG levels. Furthermore, there was no difference in the total IgG (Fig. 4A) and IgM (Fig. 4B), nor viral-specific IgG (Fig. 4C) and IgM (Fig. 4D) antibody titers between MHV68-infected CD19 Cre-positive and CD19 Cre-negative mice at 16 days post-infection.

Both EBV and MHV68 are known to drive a robust non-viral specific B-cell differentiation that leads to a rapid, though transient increase in antibody titers against self- and irrelevant (foreign) antigen (47, 48). The presence of irrelevant antibodies against horse red blood cells is diagnostic for recent EBV infection (49). In the context of MHV68 infection, global and T cell-specific IL-17RA signaling both supported the induction of irrelevant and self-directed antibodies (45, 50). To determine whether B cell-intrinsic IL-17RA signaling helps support the induction of self-directed and irrelevant antibodies, we repurposed a clinical assay used in the diagnosis of autoimmune diseases (antinuclear antibody or ANA) (48). Sera from mock and 16 day post-infected CD19 Cre-positive and CD19 Cre-negative mice were used on ANA slides coated with Hep-2 cells, which express a wide array of antigens. In contrast to what was observed in the global and T cell-specific IL-17RA-deficient models at 16 days post-infection, loss of B cell-intrinsic signaling had no impact on the MHV68-driven induction of self-directed and irrelevant antibodies as observed through ANA staining (Fig. 4E and F) and anti-double-stranded DNA (dsDNA)-directed antibodies (Fig. 4G).

At 42 days post-infection, representing long-term latency, the germinal center response continues to be reduced in CD19 Cre-positive mice (Fig. 3J through M). Despite this persistent attenuation, we do not find any measurable differences in antibody responses between infected CD19 Cre-positive and CD19 Cre-negative mice (Fig. 4H through L). Taken together, B cell-intrinsic IL-17RA signaling has no impact on the general, viral-specific, or the self-directed and irrelevant antibody response driven by MHV68 infection.

### B cell-intrinsic IL-17RA signaling during MHV68 infection modulates some splenic B-cell populations

**Fig 5:**
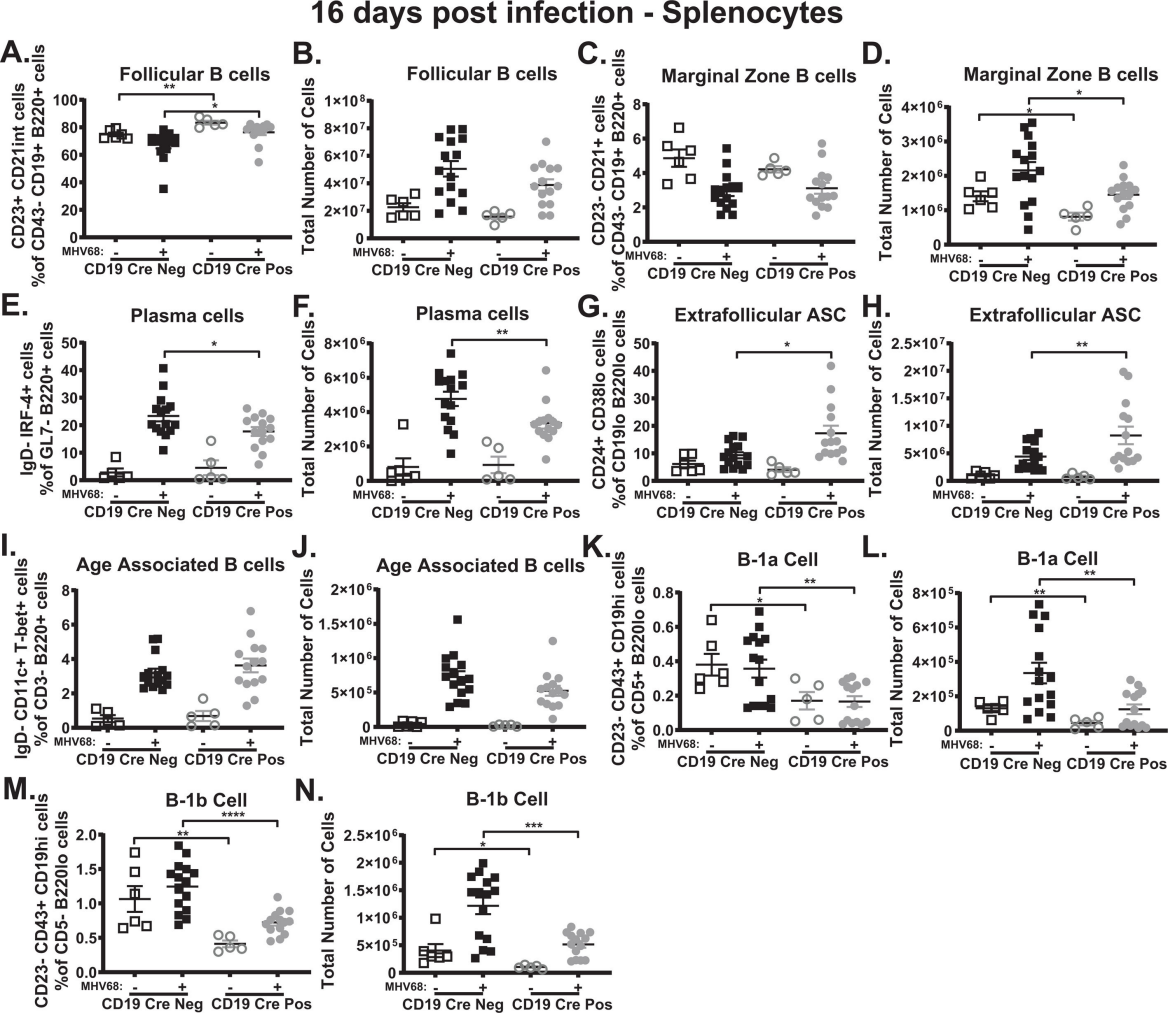
B cell-intrinsic IL-17RA signaling during MHV68 infection modulates some splenic B-cell populations. CD19 Cre-negative and CD19 Cre-positive mice were infected as in Fig. 2. The frequency (**A, C, E, G, I, K, M**) and total number (**B, D, F, H, J, L, N**) of follicular B cells, marginal zone B cells, plasma cells, extrafollicular antibody-secreting B cells, age-associated B cells, B-1a, and B-1b were determined in the spleen at 16 days post-infection. Follicular B cells were defined as B220+CD19+CD43-CD23+CD21int cells (**A, B**), marginal zone B cells were defined as B220+CD19+CD43-CD23-CD21+ cells (**C, D**), plasma cells were defined as B220+GL7-IgD-IRF4+ cells (**E, F**), extrafollicular antibody-secreting B cells were defined as B220loCD19loCD24 + CD38 lo expressing cells (**G, H**) age-associated B cells were defined as B220+IgD-CD11c+T-bet+ cells (**I, J**), B-1a B cells were defined as B220loCD5+CD23-CD43+CD19hi cells (**K, L**), and B-1b B cells were defined as B220loCD5-CD23-CD43+CD19hi cells (**M, N**). Each experimental group consists of three to seven animals, and data are pooled from three independent experiments, with each symbol representing an individual mouse. Mean and standard error of the mean are shown. *P < 0.05, **P < 0.01, ***P < 0.001 ****P < 0.0001.

Given the overall reduction in splenomegaly (Fig. 3A), total B cells (Fig. 3B and C), and the germinal center response (Fig. 3D through I) in the spleen, along with no appreciable difference in the antibody response in MHV68-infected CD19 Cre-positive mice compared to CD19 Cre-negative mice (Fig. 4), the impact of B cell-intrinsic IL-17RA signaling on B-cell populations during MHV68 infection was determined at 16 days post-infection. Figure S3 shows all gating strategies used to obtain frequencies shown in Fig. 5.

Follicular and marginal zone B cells were first examined with loss of B cell-intrinsic IL-17RA, causing an increase in the frequency (Fig. 5A), but not number (Fig. 5B) of follicular B cells in naïve and MHV68-infected mice. No significant difference was observed in the frequency of marginal zone B cells (Fig. 5C) between naïve and MHV68-infected CD19 Cre-positive and CD19 Cre-negative mice. There was, however, a statistical reduction in the total number of marginal zone B cells (Fig. 5D) between naïve and MHV68-infected CD19 Cre-positive and CD19 Cre-negative mice.

Next, antibody-secreting cells were examined. There was a significant reduction in the frequency (Fig. 5E) and number (Fig. 5F) of plasma cells in MHV68-infected CD19 Cre-positive compared to CD19 Cre-negative mice. Interestingly, loss of B cell-intrinsic IL-17RA expression during MHV68 infection led to a significant increase in the frequency (Fig. 5G) and number (Fig. 5H) of extrafollicular antibody-secreting cells (66). Age-associated B cells also produce antibodies (67), and no difference in the frequency (Fig. 5I) or number (Fig. 5J) of age-associated B cells was observed in the spleen between naïve and MHV68-infected CD19 Cre-positive and CD19 Cre-negative mice. Finally, B-1 cells, which secrete natural antibodies (68, 69) and can be subdivided into B-1a and B-1b cells on the basis of CD5 expression (70), were examined. Loss of IL-17RA signaling on B cells resulted in a significant reduction in the frequency (Fig. 5K and M) and number (Fig. 5L and N) of B-1a and B-1b B cells in naïve and MHV68-infected CD19 Cre-positive mice compared to CD19 Cre-negative mice. Taken together, these data suggest that B cell-intrinsic IL-17RA signaling not only promotes the germinal center response (Fig. 3) and subsequent plasma cell differentiation (Fig. 5E and F), but it also restricts the expansion of extrafollicular antibody-secreting B cells (Fig. 5G and H). Furthermore, B cell-intrinsic IL-17RA contributes to the expansion of B-1a (Fig. 5K and L) and B-1b (Fig. 5M and N) B-1 cell subsets in naïve mice and following MHV68 infection.

### B cell-intrinsic IL-17RA signaling during MHV68 infection supports infection of germinal center B cells

Having observed reduced latency in the spleen as well as a decreased MHV68-driven germinal center response in CD19 Cre-positive mice, the efficiency of germinal center B-cell infection was examined next, given that germinal center B cells contain a majority of the splenic latent viral reservoir at 16 days post-infection. The MHV68.ORF73bla reporter virus, which expresses a fused mLANA-b-lactamase protein in latently infected cells (71, 72), was used to label latently infected cells by flow cytometry using a cell-permeable b-lactamase substrate (CCF2). There were significantly fewer CD19 Cre-positive splenic B cells harboring MHV68 latent virus compared to CD19 Cre-negative mice (8.2-fold decrease) (Fig. 5A and B), which is in line with the observed attenuation of splenic latency in the CD19 Cre-positive mice (Fig. 2A).

There was also a significant decrease (8.2-fold) in the frequency of MHV68 latently infected germinal center B cells from CD19 Cre-positive mice compared to MHV68 latently infected germinal center B cells from CD19 Cre-negative mice (Fig. 5C and D). Taken together, these data indicate that B cell-intrinsic IL-17RA signaling not only promotes the MHV68-driven germinal center response (Fig. 3), but it also supports MHV68 latent infection of germinal center B cells as the attenuation of the germinal center response alone (~2-fold decrease) in MHV68-infected CD19 Cre-positive does not fully account for the over eightfold decrease in MHV68 latently infected cells in the spleen.

### B cell-intrinsic IL-17RA signaling in the peritoneal cavity during MHV68 infection promotes B-cell latency and expansion of B-1 B cells

The impact of B cell-intrinsic IL-17RA signaling on the peritoneal cavity during MHV68 infection was examined next. Loss of B cell-intrinsic IL-17RA signaling during MHV68 infection resulted in approximately 1 million fewer peritoneal cells on average. That reduction in total peritoneal cells was not statistically significant (Fig. 7A). To determine the impact loss of B cell-intrinsic IL-17RA signaling had on the distribution of latently infected cells in the peritoneal cavity at 16 days post-infection, given the decrease in overall viral latency observed there (Fig. 2C) in the absence of B cell-intrinsic IL-17RA signaling, we magnetically sorted CD19-positive B cells from MHV68-infected CD19 Cre-positive and CD19 Cre-negative mice and performed limiting dilution-PCR to determine the frequency of latently infected peritoneal B cells (Fig. 7B) and non-B cells (Fig. 7C). B cells lacking IL-17RA harbored significantly less latent MHV68 (Fig. 7B), while there was no difference in the frequency of MHV68 latency in non-B cells between CD19 Cre-positive and CD19 Cre-negative mice (Fig. 7C).

In the peritoneal cavity, macrophages and B-1 B cells make up the majority of the latent viral reservoir (59, 60, 73, 74). Loss of IL-17RA B cell-intrinsic signaling had no impact on the frequency of CD11b+ macrophages (Fig. 7D), yet there were significantly fewer overall CD11b+ macrophages in MHV68-infected CD19 Cre-positive mice compared to CD19 Cre-negative mice (Fig. 7E). Looking at B cells, there was no difference in the frequency (Fig. 7F) or total number (Fig. 7G) of B220+ B cells in the peritoneal cavity of naïve or MHV68-infected CD19 Cre-positive and CD19 Cre-negative mice. B-1 cells are the predominant B cells to foster latent infection in the peritoneal cavity and are subdivided into B-1a (CD5 neg) and B-1b (CD5 pos) cells based on CD5 expression, with B-1b cells harboring most, if not all, of the latent MHV68 compared to B-1a cells (59, 60). Unlike what was observed in the spleen at 16 days post-infection (Fig. 5K through N), there was no significant difference in the frequency (Fig. 7H and J) or total number (Fig. 7I and K) of B-1a and B-1b cells in naïve CD19 Cre-positive and CD19 Cre-negative mice. Upon MHV68 infection, there is a significant reduction in the frequency (Fig. 7H and J) and total number (Fig. 7I and K) of B-1a and B-1b cells in CD19 Cre-positive mice compared to CD19 Cre-negative mice. Taken together, these data indicate that B cell-intrinsic IL-17RA signaling supports the establishment of viral latency in B cells as well as the expansion of both B-1 B cell subsets during MHV68 infection in the peritoneal cavity. Figure S4 shows the gating schemes for the determination of all reported cellular frequencies in the peritoneal cavity.

## DISCUSSION

In this study, we uncovered the proviral role of B cell-intrinsic IL-17RA signaling during MHV68 infection through the generation of a B cell-specific IL-17RA-deficient mouse model. Loss of IL-17RA signaling in B cells during MHV68 infection led to an attenuation of viral latency and reactivation and suppression of B-1 cell populations in both the spleen and peritoneal cavity, as well as a significant reduction in the MHV68-driven germinal center response in the spleen. These data indicate that B cell-intrinsic IL-17RA signaling helps facilitate the establishment of chronic MHV68 infection, supports the MHV68-driven germinal center response, and expansion of B-1 cell populations.

### B cell-intrinsic function of IL-17RA signaling during MHV68 infection

Our previous studies were the first to demonstrate that global IL-17RA signaling and T cell-intrinsic IL-17RA signaling are proviral in the establishment of chronic MHV68 infection (45, 50). Those previous studies found that both global and T cell-intrinsic IL-17RA signaling supported the establishment of viral latency, viral reactivation, and the MHV68-driven germinal center response. As EBV and MHV68 are B-cell-tropic viruses (7–11), we set out to better understand the impact B cell-intrinsic IL-17RA signaling had on MHV68 infection. Loss of IL-17RA signaling in B cells was sufficient to attenuate MHV68 latency and reactivation in the spleen (Fig. 2A and B), and unlike what was observed in the T cell-specific deficiency (50), in the peritoneal cavity as well (Fig. 2C and D). The decrease in viral reactivation and latency in the peritoneal cavity is similar to what was observed in the global IL-17RA-deficient mouse model (45).

Loss of B cell-intrinsic IL-17RA signaling during MHV68 infection led to a reduction in overall spleen size following infection (Fig. 3A). Furthermore, there were significantly fewer B220+ B cells in the spleen of mice lacking B cell-intrinsic IL-17RA signaling during MHV68 infection (Fig. 3B and C). Similar to what was observed in the global and T cell-deficient IL-17RA signaling models (45, 50), loss of B cell-specific IL-17RA signaling also significantly attenuated the MHV68-driven germinal center response at 16 days post-infection (Fig. 3D through I). The germinal center response was still attenuated with the loss of IL-17RA B cell-intrinsic signaling at 42 days post-infection (Fig. 3J through M). These findings indicate that B cell-intrinsic IL-17RA signaling is also important in sustaining the germinal center response during long-term latent MHV68 infection. Given that both B cell-intrinsic IL-17RA signaling (Fig. 3) and T cell-intrinsic IL-17RA signaling (50) alone supported the expansion of MHV68-driven germinal center, while impacting signaling in only one cell type, either germinal center B cells or the T follicular helper cells, highlights the interconnectedness of germinal center B cells and T follicular helper cells in the germinal center response. If either the expansion of germinal center B cells or T follicular helper cells is restricted due to lack of IL-17RA signaling in one of those cell types, then we get a similar reduction in the other cell type as they work together to support the expansion of the germinal center response.

**Fig 6:**
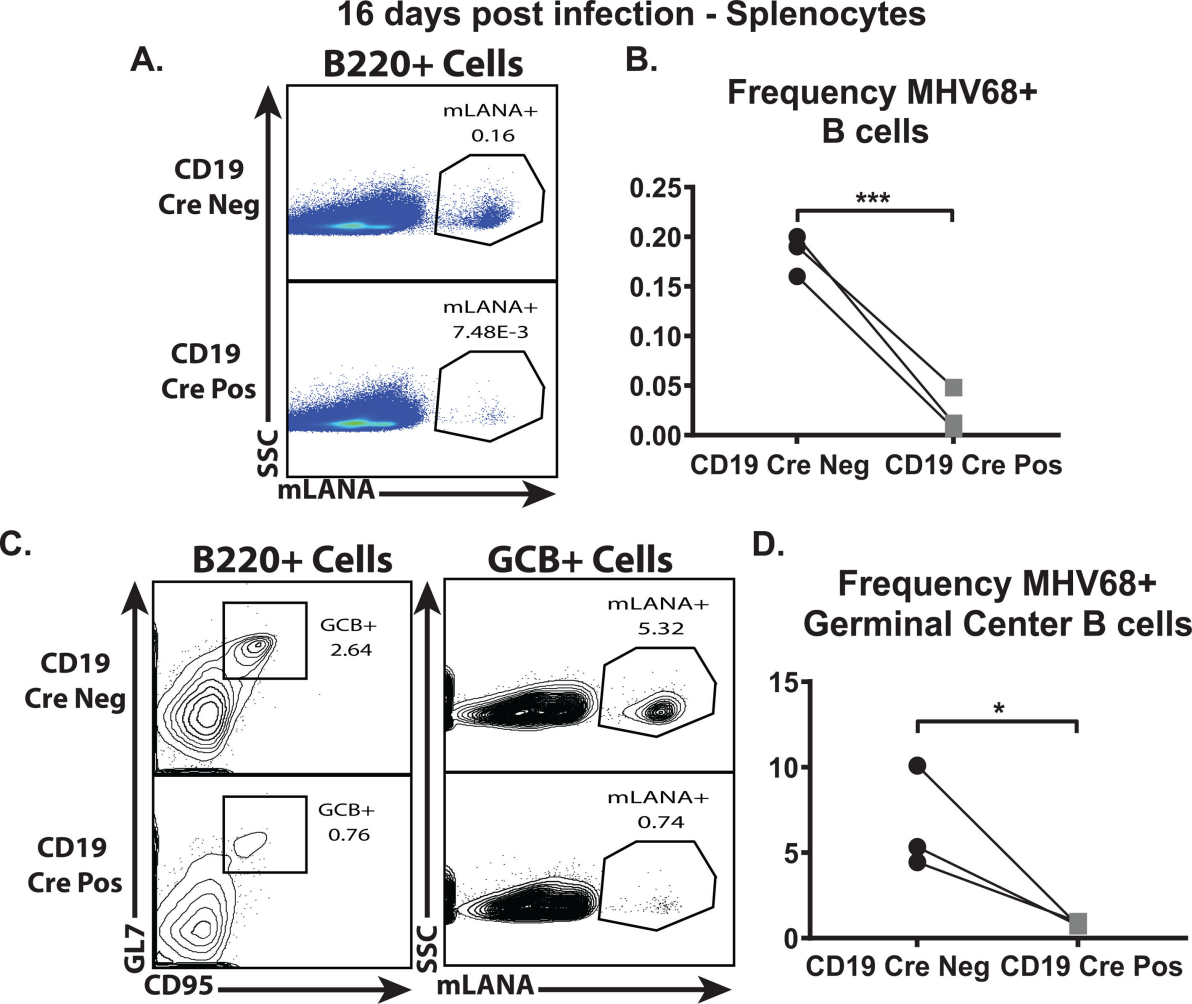
B cell-intrinsic IL-17RA signaling during MHV68 infection supports infection of germinal center B cells. CD19 Cre-negative and CD19 Cre-positive mice were intranasally infected with 10,000 PFU of MHV68.ORF73βla. At 16 days post-infection, splenocytes were isolated, pooled within each group, and stained for surface markers B220, CD95, and GL7. The cells were then loaded with CCF2-AM, the fluorescent β-lactamase substrate, for 1 hour at room temperature. Pooled splenocytes were examined for mLANA expression (β-lactamase activity) via flow cytometry, as indicated by the fluorescent signal of cleaved CCF2-AM (mLANA+). (**A**) Representative gating strategy for B220+ mLANA+ cells in the spleen. (**B**) The frequency of mLANA+ B cells, as defined by B220+, mLANA+ cells. (**C**) Representative gating strategy for germinal center B cells (B220+ CD95+ GL7+), then gated for mLANA+ cells in the spleen. (**D**) The frequency of mLANA+ germinal center B cells, as defined by B220+ CD95+ GL7+, mLANA+ cells. Data are representative of three independent experiments with a minimum of three to four mice pooled per genotype (CD19 Cre negative and CD19 Cre positive) per experiment. Lines represent paired observations within a single experiment. *P < 0.05, ***P < 0.001.

To determine whether attenuation (twofold reduction) of the germinal center response in IL-17RA-deficient B cells (Fig. 3) was solely responsible for the reduced MHV68 latency in the spleen (Fig. 2A), the MHV68.ORF73bla reporter virus was utilized. Lack of IL-17RA signaling in B cells resulted in an eightfold reduction in the frequency of MHV68-infected germinal center B cells (Fig. 6C and D). The frequency of germinal center B cells was only reduced twofold in infected B cell-specific IL-17RA-deficient mice compared to IL-17RA-sufficient mice, which indicates that IL-17RA signaling in B cells not only supports the MHV68-driven germinal center response, but it also helps facilitate the establishment of latency in germinal center B cells.

Interestingly, loss of B cell-specific IL-17RA signaling had no impact on the humoral response to MHV68 infection at 16 and 42 days post-infection (Fig. 4), despite the attenuated germinal center response (Fig. 3). This was surprising, particularly for irrelevant and self-directed antibody production, given that global and T cell-specific deficient IL-17RA signaling loss resulted in a significant defect in the generation of irrelevant and self-directed antibodies (45, 50), a process that is unique to gammaherpesvirus infection (47, 48). These data indicate that the irrelevant and self-directed antibody response during gammaherpesvirus infection driven by IL-17RA signaling is T cell-dependent, as only when IL-17RA signaling is lost in T cells do we see the attenuation of these responses (45, 50). Future studies will examine the T cell-dependent role of IL-17RA signaling in promoting the irrelevant and self-directed antibody response during gammaherpesvirus infection.

**Fig 7:**
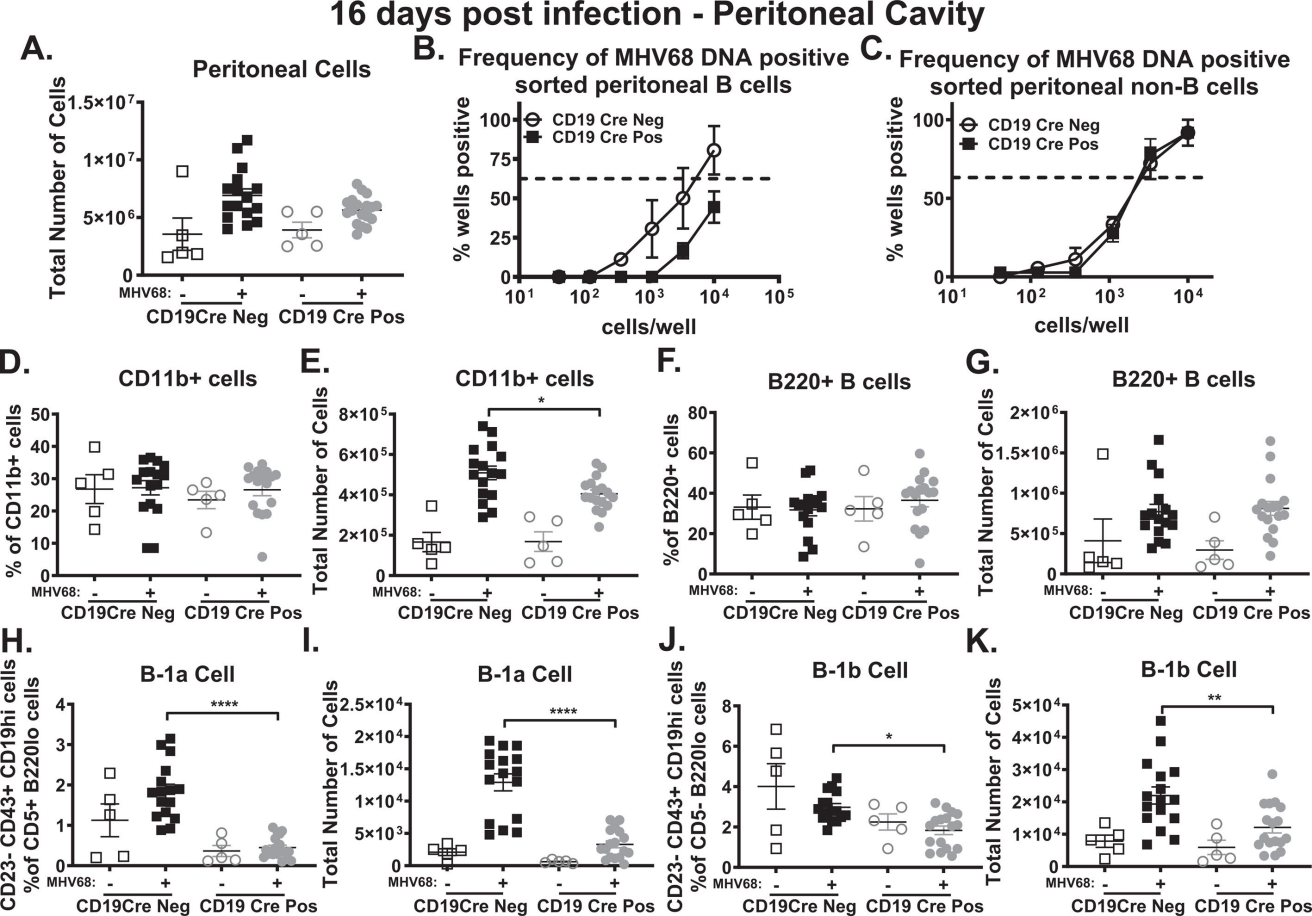
B cell-intrinsic IL-17RA signaling in the peritoneal cavity during MHV68 infection promotes B-cell latency and expansion of B-1 B cells. CD19 Cre-negative and CD19 Cre-positive mice were infected as in Fig. 2. Total peritoneal cells from naïve and infected CD19 Cre-negative and CD19 Cre-positive mice at 16 days post-infection (**A**). At 16 days post-infection, peritoneal cells were pooled from infected CD19 Cre-negative and CD19 Cre-positive mice with 5–6 mice per group in each experiment. Pooled peritoneal cells were magnetically sorted into CD19+ B cells (**B**) and CD19− non-B cells (**C**), with both populations being subject to a limiting dilution PCR assay. Peritoneal cells from the individual-infected mice were subject to flow cytometry. The frequency (**D, F, H, J**) and total number (**E, G, I, K**) of CD11b+ macrophages (**D,E**), B220+ B cells (**F, G**), B-1a (**H, **I), and B-1b (**J, K**) B cells. B-1a B cells were defined as B220loCD5+CD23-CD43+CD19hi cells (**H, I**), and B-1b B cells were defined as B220loCD5-CD23-CD43+CD19hi cells (**J, K**). Each experimental group consists of three to seven animals, and data are pooled from three independent experiments, with each symbol representing an individual mouse. Mean and standard error of the mean are shown. *P < 0.05, **P < 0.01, ****P < 0.0001.

One potential explanation as to why there was no difference in the humoral response in the absence of B cell-intrinsic IL-17RA signaling during gammaherpesvirus infection is the role B cell-intrinsic IL-17RA signaling has in modulating B-cell populations. Loss of B cell-intrinsic IL-17RA signaling resulted in a significant decrease in the frequency and number of plasma cells in MHV68-infected mice (Fig. 5E and F). Interestingly, loss of B cell-intrinsic IL-17RA signaling increased the frequency and number of extrafollicular antibody-secreting B cells (Fig. 5G and H). Age-associated B cells also produce antibodies (67), and loss of IL-17RA signaling in B cells had no impact on the age-associated B cells during MHV68 infection (Fig. 5I and J). B-1 cells are a group of B cells that produce natural antibodies (68, 69) and loss of IL-17RA signaling in B cells resulted in significant reductions in both B-1a (Fig. 5K and L) and B-1b (Fig. 5M and N) subsets of B-1 B cells in naïve and MHV68-infected mice in the spleen. In the peritoneal cavity, a significant reduction in both B-1a (Fig. 7H and I) and B-1b (Fig. 7J and K) was only observed following MHV68 infection in the CD19 Cre-positive mice compared to CD19 Cre-negative mice. Loss of IL-17RA signaling in B cells during MHV68 infection resulted in a loss of around 50%–60% of the plasma cells (Fig. 5F) and B-1a (Fig. 5L) and B-1b (Fig. 5N) cells in the spleen. The loss of antibody-secreting plasma cells (Fig. 5F) and B-1a (Fig. 5L) and B-1b (Fig. 5N) cells was accounted for by a nearly 100% increase in extrafollicular antibody-secreting B cells (Fig. 5H) in MHV68-infected mice lacking B cell-intrinsic IL-17RA signaling. These data indicate that despite a decrease in B-1a and B-1b cells and a reduced germinal center response leading to fewer plasma cells in the absence of B cell-intrinsic IL-17RA signaling during MHV68 infection, the loss of those antibody-secreting cells is made up for in an increase in extrafollicular antibody-secreting cells. Furthermore, our data demonstrate that B cell-intrinsic IL-17RA signaling not only supports the germinal center response (Fig. 3) and differentiation of plasma cells (Fig. 5E and F); it restricts the expansion of extrafollicular antibody-secreting B cells (Fig. 5G and H) during MHV68 infection, while promoting the expansion of B-1a (Fig. 5K, L, 7H and I) and B-1b (Fig. 5M, N, 7J and K) cells. To our knowledge, this is the first time IL-17RA signaling has been implicated in impacting extrafollicular antibody-secreting B cells. Our data also support and expand on findings by Wang et al. (38) that found IL-17RA signaling promotes B-1a cell differentiation and natural antibody production in the lungs during influenza virus infection (38). We show that IL-17RA signaling in B cells also promotes B-1b B cell (Fig. 5M, N, 7J and K) activation in addition to B-1a B cell (Fig. 5K, L, 7H and I) differentiation in naïve mice in the spleen and during MHV68 infection in the spleen and peritoneal cavity.

Loss of IL-17RA signaling in B cells during MHV68 infection also led to significant attenuation in viral latency (Fig. 2C) and reactivation (Fig. 2D) in the peritoneal cavity. To determine how B cell-intrinsic IL-17RA signaling supports the establishment of latency in the peritoneal cavity, we examined viral latency in B cells (Fig. 7B) and non-B cells (Fig. 7C). Loss of IL-17RA signaling in B cells during MHV68 infection only impacted viral latency in B cells (Fig. 7B) with viral latency in non-B cells being identical in the presence or absence of IL-17RA signaling in B cells (Fig. 7C). Viral latency in non-B cells is primarily in macrophages (73, 74), while B-1b cells make up the majority of peritoneal B cells harboring latent virus (59, 60). Loss of B cell-intrinsic IL-17RA signaling during MHV68 infection led to a reduction in the number of CD11b+ macrophages (Fig. 7E) as well as the frequency and number of B-1a (Fig. 7H and I) and B-1b (Fig. 7J and K). Taken together, one can see how despite no difference in the frequency of viral latency in macrophages (Fig. 7C) in the absence of B cell-intrinsic IL-17RA signaling during MHV68 infection, there is an overall decrease in viral latency in the absence of IL-17RA signaling in B cells in the peritoneal cavity given the fewer macrophages (Fig. 7E) and significantly fewer B-1b B cells (Fig. 7K). These data indicate that B cell-intrinsic IL-17RA signaling during MHV68 infection supports the establishment of latency in peritoneal B cells (Fig. 7B) as well as the expansion of B-1a (Fig. 7H and I) and B-1b (Fig. 7J and K) B cells.

This study revealed that some of the proviral effects observed in global IL-17RA signaling deficiency during MHV68 infection (45) can be attributed to B cell-intrinsic IL-17RA signaling. B cell-intrinsic IL-17RA signaling supported the establishment of MHV68 latency and viral reactivation in the spleen and peritoneal cavity (Fig. 2). It helps promote the MHV68-driven germinal center response (Fig. 3) and supports the establishment of MHV68 latency in germinal center B cells (Fig. 6) and peritoneal cavity B cells (Fig. 7B), all of which were phenotypes observed in the global IL-17RA-deficient model (45). When comparing MHV68-dependent phenotypes between the global-deficient, T cell-deficient, and now B cell-deficient IL-17RA signaling models, it is clear that multiple cell types are responsible for the proviral phenotype seen in the global-deficient model. Both B and T cell-intrinsic IL-17RA signaling support the establishment of MHV68 latency and viral reactivation in the spleen (Fig. 2A and B) (50), while B cell-intrinsic signaling supports the establishment of MHV68 latency and viral reactivation in the peritoneal cavity (Fig. 2C and D). Both help support the MHV68-driven germinal center response (Fig. 3), while T cell-intrinsic (50), but not B cell-intrinsic (Fig. 4), IL-17RA signaling helps support the generation of irrelevant and self-directed antibodies. In the future, studies will continue to determine the combination of cell subsets that require IL-17RA signaling to support MHV68 infection as well as investigate potential mechanisms of IL-17RA signaling that support MHV68 infection.

### B cell-intrinsic IL-17RA signaling function in the B-cell responses and establishment of MHV68 latency

There are multiple ways in which B cell-intrinsic IL-17RA signaling may help promote the establishment of MHV68 latency. Previous reports have shown that IL-17RA signaling helps promote B-cell activation (52) and, importantly, germinal center formation and migration (53–57) in the context of autoimmunity and bacterial infection. Furthermore, IL-17RA signaling via Blimp-1 expression and NF-kB activity in B-1a cells promotes differentiation and natural antibody production during pulmonary influenza virus infection (38). Both B-cell activation and germinal center formation, which IL-17RA signaling can affect (52–58), are critical for the establishment of latent gammaherpesvirus infection (7–9, 12). We found that loss of B cell-intrinsic IL-17RA signaling in the context of MHV68 infection led to attenuated MHV68 latency (Fig. 2 and 6) and MHV68-driven germinal center response (Fig. 3) in the spleen. The reduction in the MHV68-driven germinal center response was not solely responsible for the full attenuation of MHV68 latency in the spleen (Fig. 6). One potential way in which B cell-intrinsic IL-17RA signaling could promote the MHV68-driven germinal center response is through activation of NF-kB downstream of the receptor (27–29). NF-kB activity has been shown to support the establishment of MHV68 latency and the germinal center response during MHV68 infection (75–77). Attenuation of NF-kB activity in the absence of IL-17RA signaling in B cells could lead to both a reduction in the MHV68-driven germinal center response and MHV68 latent viral levels. Furthermore, we observed that B cell-intrinsic IL-17RA signaling was important for the expansion of both B-1a (Fig. 5K, L, 7H and I) and B-1b (Fig. 5M, N, 7J and K) B cells in the spleen and peritoneal cavity during MHV68 infection. B-1b cells in particular are an important latent viral reservoir in the peritoneal cavity (59, 60). A previous report showed that, via Blimp-1 expression and NF-kB activity in B-1a cells, downstream of IL-17RA signaling is important for B-1a cell differentiation (38). The lack of B-1a (Fig. 5K, L, 7H and I) and B-1b (Fig. 5M, N, 7J and K) B cells expansion in the spleen and peritoneal cavity during MHV68 infection could be due to the lack of NF-kB activity downstream of IL-17RA signaling in those cells. Future studies will be done to determine if that is the mechanism driving B-1b B-cell expansion during MHV68 infection. The lack of B-1b cell expansion in the peritoneal cavity (Fig. 7H and I) is potentially due to loss of NF-kB activity downstream of IL-17RA signaling in B cells, along with the decrease in efficiency to infect B cells in the peritoneal cavity (Fig. 7B) seen during MHV68 infection in mice lacking IL-17RA signaling in B cells, which would explain the decreased viral latency observed in the peritoneal cavity (Fig. 2C). These results highlight another aspect of NF-kB activity downstream of IL-17RA signaling in B cells other than germinal center B cells, which may directly impact the establishment of MHV68 latency this time in the peritoneal cavity.

NF-kB activity is not the only potential route by which B cell-intrinsic IL-17RA signaling could promote MHV68 latency establishment. IL-17RA signaling can stimulate glucose uptake through the induction of Iκbζ (78), which can stimulate immune cell activation. Glucose metabolism can promote gammaherpesvirus infection (79–82) as well as play an important role in supporting the exponential expansion of B cells in the germinal center response (80–82) and other important B-cell functions (83, 84). IL-17RA signaling also promotes mRNA stabilization through the universally expressed RNA-binding protein HuR (also known as ELAVL1) (85–87). Loss of efficient mRNA stabilization may explain the decreased MHV68 latency efficiency observed in IL-17RA-deficient germinal center B cells (Fig. 6). Future studies will work to identify downstream signaling mechanisms of IL-17RA in B cells and other cells, which could explain its proviral role in MHV68 infection.

This study is the first, to our knowledge, to directly examine the B cell-intrinsic role of IL-17RA signaling in the context of viral infection and show a role for it in the establishment of MHV68 latent infection, the MHV68-driven germinal center response, and expansion of both B-1a and B-b B cells. Previous studies looking at the impact of IL-17RA signaling on B cells have relied on global-deficient mouse models and IL-17A or IL-17RA neutralizing antibodies. The mice generated in this study will provide an excellent tool to understand the significance of IL-17RA signaling in B cells in a wide range of autoimmune and infection models.

## MATERIALS AND METHODS

### Animals used

To generate mice with B cell-specific IL-17RA deficiency, IL-17RA^fl/fl^ (B6.Cg-IL-17ra^tm2.1Koll^/J stock 031000) mice (61) were bred to CD19 Cre-positive (B6.129P2-*Cd19^tm1(cre)Cgn^*/J) mice (62) from The Jackson Laboratories (Bar Harbor, ME). All mice were housed and bred in a specific pathogen-free facility. All experimental manipulations of mice were approved by the Institutional Animal Care and Use Committee of Western Michigan University Homer Stryker M.D. School of Medicine (2022-0026). Both male and female mice were used, with no sex-specific phenotypes noted.

### Magnetic sorting

CD19+ splenic B cells and B cells in the peritoneal cavity were negatively selected according to the manufacturer’s instructions (MojoSort Mouse CD19 B cell Isolation Kit, BioLegend, San Diego, CA).

### Western blot

Magnetically sorted CD19+ B cells from naïve CD19 Cre-negative and CD19 Cre-positive mice were used to assess IL-17RA protein expression. Sorted B cells were lysed in Laemmli buffer and subjected to Western analyses as previously described (88). The following antibodies were used: anti-IL-17RA (1:5,000), anti-β actin (1:40,000), a goat anti-rabbit horseradish peroxidase-conjugated secondary antibody (1:15,000), and a goat anti-mouse horseradish peroxidase-conjugated secondary antibody (1:15,000) (Thermo Fisher Scientific, Waltham, MA).

### Infections

Between 6 and 10 weeks of age, mice were intranasally inoculated with 1,000 PFU of MHV68 (WUMS) diluted in sterile serum-free Dulbecco’s modified Eagle’s medium (15 µL/mouse), under light anesthesia. MHV68 viral stock was prepared and titered on NIH 3T12 cells. The spleen and peritoneal cells were harvested from euthanized mock-treated and MHV68-infected animals at the indicated times post-infection. Mice were euthanized by CO_2_ inhalation from a compressed gas source in a non-overcrowded chamber. Mice were bled prior to euthanasia via a submandibular route, and serum was isolated using BD Microtainer blood collection tubes (Becton, Dickinson and Company, Franklin Lakes, NJ).

### Limiting dilution assays

Frequency of cells harboring viral DNA was determined by limiting dilution PCR analysis, while the frequency of *ex vivo* reactivation was determined by limiting dilution assay as previously described (89). Briefly, to determine the frequency of cells reactivating virus *ex vivo*, serial two-fold dilutions of splenocytes or peritoneal cell suspensions harvested from infected mice were plated onto monolayers of mouse embryonic fibroblasts (MEFs) immediately following harvest, at 24 replicates per dilution. To control for preformed infectious virus, twofold serial dilutions of mechanically disrupted splenocytes or peritoneal cells were plated as above. MHV68 was allowed to reactivate from explanted cells, and the virus was further amplified within the same well via subsequent replication in MEF. At 21 days post-plating, all replicates were scored in a binary fashion for the presence of live fibroblasts (no viral reactivation/replication) or absence of such (cytopathic effect driven by lytic replication). Because primary MEFs were used to amplify the virus, the sensitivity of the limiting dilution reactivation assay was below a single PFU of MHV68, as defined using a 3T12-based plaque assay. Because the endpoint of viral amplification in MEF was measured, the limiting dilution reactivation assay was not susceptible to variability of titers released from primary cells upon viral reactivation *ex vivo* (90).

### Flow cytometry

Single-cell suspensions of splenocytes and peritoneal cells from individual mice were prepared in FACS buffer (phosphate-buffered saline [PBS] + 2% FCS +0.05% sodium azide) at 1 × 10^7^ nucleated cells/mL. A total of 2 × 10^6^ cells were treated with Fc block (24G2) prior to extracellular staining for 30 minutes on ice. Data acquisition was performed on an Attune NxT flow cytometer (Thermo Fisher Scientific, Waltham, MA) and a Fortessa flow cytometer (BD Biosciences, San Jose, CA) and analyzed using FlowJo software (Becton, Dickinson & Company, Ashland, OR). The following antibodies were purchased from BioLegend (San Diego, CA) for use in this study: CD3 (17A2), CD4 (RM4-5), CD5 (53-7.3), CD11b (M1/70), CD11c (N418), CD19 (6D5), CD21 (7E9), CD22 (OX-97), CD23 (B3B4), CD38 (88), CD43 (S11), CD44 (IM7), CD86 (GL-1), PD-1 (29 f.1A12), B220 (RA3-6B2), GL7 (GL-7), CXCR4 (L276F12), CXCR5 (L138D7), IRF-4 (IRF4.3E4), T-bet (4B10), IgD (11-26c.2a), and IgM (RMM-1). CD95 (JO2) was purchased from BD Horizon (San Jose, CA). IL-17RA (PAJ-17R) was purchased from Thermo Fisher Scientific (Waltham, MA. Cat #12-7182-82). Compensation controls were done using OneComp eBeads (Thermo Fisher Scientific, Waltham, MA). Briefly, a negative control (beads alone) was used to establish a baseline PMT (photomultiplier tube) voltage and fluorescent background. Positive controls for each fluorochrome (beads with a single fluorochrome) were used to establish spill-over of the individual fluorochrome into the other channels being used. PMT values are adjusted for each fluorochrome to minimize spillover.

### MHV68.ORF73βla studies

Mice were infected intranasally with 10,000 PFU MHV68.ORF73βla. Mice were euthanized at 16 days post-infection, with splenocytes pooled from each group (3 mice/group). Cells (2 × 10^7^) from each group were Fc blocked and then stained with B220 and GL7 for 30 minutes on ice as described above. The cells were washed twice and loaded with CCF2-AM (GeneBLAzer kit; ThermoFisher Scientific, Waltham, MA) at room temperature for 1 hour. The cells were then washed twice and suspended in FACS buffer prior to analysis via flow cytometry.

### ELISA

Total, MHV68-specific, and dsDNA immunoglobulin levels were determined as previously described (91). Briefly, Nunc Maxisorp plates (Fisher Scientific, Pittsburgh, PA) were coated with anti-IgG (Heavy+Light) or anti-IgM antibodies (Jackson ImmunoResearch, West Grove, PA), UV-irradiated MHV68 virus stock in PBS (740,000 microjoules/cm^2^ × 2) (Stratalinker UV Crosslinker 1800; Agilent Technologies, Santa Clara, CA), or dsDNA from *Escherichia coli* (12·5 µg/mL; Sigma-Aldrich, St. Louis, MO) overnight at 4°C. Plates were washed with PBS-Tween (0.05%), blocked for 1 hour with PBS-Tween (0.05%)-BSA (3%), incubated with fivefold serial dilutions of serum in PBS-Tween (0.05%)-BSA (1.5%) for 2 hours, and washed with PBS-Tween (0.05%). Bound antibody was detected with horseradish peroxidase-conjugated goat anti-mouse total IgG (heavy + light chain) or IgM (Jackson ImmunoResearch, West Grove, PA) using 3,3′, 5,5′-tetramethylbenzidine substrate (Life Technologies, Gaithersburg, MD). HRP enzymatic activity was stopped by the addition of 1 N HCl (Sigma-Aldrich, St. Louis, MO), and the absorbance was read at 450 nm on BioTek EPOCH2 Plate Reader (Agilent Technologies, Santa Clara, CA).

### ANA panels

Antinuclear antibodies were assessed with an Antinuclear Antibody (ANA) Test Kit (Antibodies Inc., Davis, CA). Following the manufacturer’s protocol, serum was diluted (1:40 in PBS) and incubated over slides coated with fixed HEp-2 cells. Following serum incubation, the slides were rinsed and stained with anti-mouse IgG Alexa Fluor-488 (H+L) (ThermoScientific, Waltham, MA). Fluorescent images were captured using NIS Elements software. Corrected fluorescence was quantified using ImageJ software from a randomly chosen field of ~10 cells in each sample.

### Statistical analyses

Statistical analyses were performed using Student’s *t* test when comparing two groups and one-way ANOVA with Tukey’s post hoc test when comparing more than two groups (Prism, GraphPad Software, Inc.).

## Data Availability

All data associated with this study are presented in the figures.

## SUPPLEMENTAL MATERIAL

Fig. S1 jvi.01842-25-s0001.eps Frequency and absolute number of CD3+ T cells and CD11b+ monocytes in the spleens of CD19 Cre negative and CD19 Cre positive naïve littermates.

Fig. S2 jvi.01842-25-s0002.eps Frequency and absolute number of B220+GL7+CD95+CXCR4+CD86- dark zone centroblasts and B220+GL7+CD95+CXCR4-CD86+ light zone centrocytes in the spleens of CD19 Cre negative and CD19 Cre positive littermates.

Fig. S3 jvi.01842-25-s0003.eps Flow cytometry gating strategies in splenocytes.

Fig. S4 jvi.01842-25-s0004.eps Flow cytometry gating strategies in peritoneal cavity.

Supplemental legends jvi.01842-25-s0005.docx Legends for Fig. S1 to S4.

ASM does not own the copyrights to Supplemental Material that may be linked to, or accessed through, an article. The authors have granted ASM a non-exclusive, world-wide license to publish the Supplemental Material files. Please contact the corresponding author directly for reuse.
