# Supplementary material for: B cell-intrinsic interleukin 17 receptor A signaling supports the establishment of chronic murine gammaherpesvirus 68 infection
Source: J Virol. 2025 Dec 3;99(12):e01842-25. doi: 10.1128/jvi.01842-25 (PMC12724197; doi:10.1128/jvi.01842-25)
Supplement: Supplemental legends — Legends for Fig. S1 to S4. [file jvi.01842-25-s0005.docx]

**Supplemental Figure Legends:**

**Supplemental Figure 1.** Frequency (**A&C**) and absolute number (**B&D**) of CD3+ T cells (**A,B**) and CD11b+ monocytes (**C,D**) in the spleen of CD19 Cre negative and CD19 Cre positive naïve littermates. Data are pooled from 4-6 independent experiments, with each symbol representing an individual mouse.

**Supplemental Figure 2.** Frequency (**A&C**) and absolute number (**B&D**) of B220+GL7+CD95+CXCR4+CD86- dark zone centroblasts (**A,B**) and B220+GL7+CD95+CXCR4-CD86+ light zone centrocytes (**C,D**) in the spleen of CD19 Cre negative and CD19 Cre positive littermates. Data are pooled from 2 independent experiments, with each symbol representing an individual mouse. Mean and standard error of the mean are shown. *p<0.05

**Supplemental Figure 3.** Flow cytometry gating strategies in splenocytes. Samples to be analyzed via flow cytometry were prepared and stained as described in Materials and Methods. Relevant cell populations in the spleen of naïve and MHV68 infected CD19 Cre negative and CD19 Cre positive mice were gated as follows. Singlets and lymphocytes were gated prior to gating CD3+ T cells, CD3- cells, and B220+ cells. CD3+ T cells were then gated into CD4+ T cells and then PD-1+CXCR5+ T follicular helper cells (Tfh). B220+ cells were used to identify multiple B cell subpopulations as follows. Germinal center B cells (GCB) were defined as B220+ GL7+ CD95+ cells. Those cells were then gated further to identify B220+GL7+CD95+CXCR4+CD86- dark zone centroblasts (CB) and B220+GL7+CD95+CXCR4-CD86+ light zone centrocytes (CC). B220+ B cells were gated along with CD19 to identify CD19lo/B220lo expressing B cells, which were further gated to define extrafollicular antibody secreting B cells (EF ASC) as B220loCD19loCD24+CD38lo expressing cells. B220+ cells were further gated on CD19+ CD43- to identify B220+CD19+CD43-CD23+CD21int follicular B cells (FO) and B220+CD19+CD43-CD23-CD21+ marginal zone B cells (MZ). B220+ cells were also gated on CD19+ alone followed by IgD with GL7 to define B220+GL7-IgD-IRF4+ Plasma Cells. Those CD19+ cells were also gated on IgD- alone followed by CD11c and T bet to identify B220+IgD-CD11c+T-bet+ age associated B cells (ABC). Then using the CD3- gate, B220+ cells were gated followed by CD5 with B220 to identify B220lo expressing cells with or without CD5. Using CD5 expression as a differentiation marker, we further examined CD19 and CD23 expression followed by CD43 and CD19 expression to define B-1a B cells as B220loCD5+CD23-CD43+CD19hi cells and B-1b B cells as B220loCD5-CD23-CD43+CD19hi cells.

**Supplemental Figure 4.** Flow cytometry gating strategies in peritoneal cavity. Samples to be analyzed via flow cytometry were prepared and stained as described in Materials and Methods. Relevant cell populations in the peritoneal cavity of naïve and MHV68 infected CD19 Cre negative and CD19 Cre positive mice were gated as follows. Singlets and lymphocytes were gated prior identifying CD3- cells, B220+ cells, and CD11b+ cells. Then using the CD3- cells, B220+ cells were gated followed by CD5 with B220 to identify B220lo expressing cells with or without CD5. Using CD5 expression as a differentiation marker, we further examined CD19 and CD23 expression followed by CD43 and CD19 expression to define B-1a B cells as B220loCD5+CD23-CD43+CD19hi cells and B-1b B cells as B220loCD5-CD23-CD43+CD19hi cells.
